# Supplementary material for: High Prevalence of Atrial Fibrillation in a Lithuanian Stroke Patient Cohort
Source: Medicina (Kaunas). 2022 Jun 14;58(6):800. doi: 10.3390/medicina58060800 (PMC9230037; doi:10.3390/medicina58060800)
Supplement: Supplementary file 1 [file medicina-58-00800-s001.zip › medicina-1739494-supplementary.pdf]

Table S1. Demographic, clinical characteristics, and outcomes for stroke patients with pre-existing atrial fibrillation, and atrial fibrillation detected after stroke (AFDAS).

	**Pre-existing AF before stroke onset (n = 68)**	**AFDAS (n = 29)**	***P* value**
**Female, n (%)**	39 (57.4)	18 (62.1)	0.666
**Mean age, years (SD)**	74.6 (11.1)	78.3 (10.5)	0.122
**Baseline median mRS ≤ 2, n (%)**	63 (92.6)	25 (86.2)	0.444
**Baseline NIHSS, median (IQR)**	8.5 (5.75–16.25)	10 (6–16)	0.959
**Risk factors, n (%)**			
**Hypertension**	67 (98.5)	27 (93.1)	0.212
**Diabetes mellitus**	23 (33.8)	6 (20.7)	0.196
**Dyslipidemia**	50 (73.5)	24 (82.8)	0.514
**History of stroke/TIA**	22 (32.4)	3 (10.3)	**0.024**
**Congestive heart failure**	45 (66.2)	13 (44.8)	**0.050**
**Coronary artery disease**	38 (55.9)	14 (48.3)	0.492
**Peripheral artery disease**	5 (7.4)	0 (0.0)	0.317
**Underlying malignancy**	3 (4.4)	1 (3.4)	1
**CTA performed, n (%)**	50 (73.5)	17 (58.6)	0.146
**Reperfusion, n (%)**	27 (39.7)	11 (37.9)	
**Not eligible**	41 (60.3)	18 (62.1)	0.870
**IVT**	7 (10.3)	6 (20.7)	0.169
**EVT**	16 (23.5)	4 (13.8)	0.412
**Combined treatment**	4 (5.9)	1 (3.4)	1
**Large vessel occlusion, n (%)** †	34 (68.0)	10 (58.8)	0.491
**Anterior**	27 (39.7)	10 (34.5)	0.230
**Posterior**	5 (7.4)	0 (0.0)	0.319
**Both**	2 (2.9)	0 (0.0)	1
**None**	16 (50.0)	7 (65.5)	0.160
**Case fatality, n (%)**			
**In-hospital case fatality**	9 (13.2)	4 (13.8)	1
**90-day case fatality**	19 (27.9)	12 (41.4)	0.194
**1-year case fatality**	28 (41.2)	12 (41.4)	0.985
**Median mRS ≤ 2 at 90 days, n (%)** ‡	17 (50.0)	10 (71.4)	0.174
**Missing mRS, n (%)**	15 (30.6)	3 (17.6)	0.361
**EQ-5D domain, n (%)** ‡			
**Decreased mobility**	16 (48.5)	1 (7.1)	**0.008**
**Difficulty with self-care**	19 (57.6)	4 (28.6)	0.111
**Problems performing usual activities**	22 (68.8)	11 (78.6)	0.496
**Pain or discomfort**	16 (50.0)	5 (35.7)	0.371
**Anxious or depressed**	14 (45.2)	4 (28.6)	0.343
**EQ-5D score index, median (IQR)** ‡	0.57 (0.22–0.81)	0.75 (0.59–0.85)	0.127
**Missing EQ-5D, n (%)**	18 (36.7)	3 (17.6)	0.227

**EQ-VAS, median (IQR) ‡**	42.5 (22.5–57.5)	70 (50–75)	**0.004**
**Missing EQ-VAS, n (%)**	19 (38.8)	8 (47.0)	0.141

AF – atrial fibrillation, AFDAS – atrial fibrillation detected after stroke, SD – standard deviation, mRS – modified Rankin Scale, NIHSS – National Institutes of Health Stroke Scale, IQR – interquartile range, TIA – transient ischemic attack, CTA – computed tomography angiography, IVT – intravenous thrombolysis, EVT – endovascular treatment, EQ-5D – EuroQoL Five Dimensions, EQ-VAS – EuroQoL visual analog scale.

† Out of those in whom CTA was performed.

‡ Out of those alive at 90 days, not lost to follow-up.

Table S2. Unadjusted and adjusted binary logistic regression analysis for good functional outcome (mRS ≤ 2) as dependent variable in patients with stroke.

**Covariates**		**Unadjusted**		**Adjusted †**	
		**OR (95% CI)**	***P* value**	**OR (95% CI)**	***P* value**
**Age**		0.98 (0.95–1.01)	0.125		
**Gender**	**Female**	1.00 (reference)			
	**Male**	1.29 (0.64–2.59)	0.475		
**Atrial fibrillation**	**No**	1.00 (reference)			
	**Yes**	0.86 (0.42–1.76)	0.673		
**Reperfusion treatment**	**No**	1.00 (reference)		1.00 (reference)	
	**Yes**	1.86 (0.91–3.85)	0.091	3.91 (1.66–10.05)	**0.002**
**Baseline NIHSS**		0.87 (0.79–0.94)	**<0.001**	0.82 (0.73–0.90)	**<0.001**

OR – odds ratio, CI – confidence interval, mRS – modified Rankin Scale, NIHSS – National Institutes of Health Stroke Scale.
† Akaike information criterion = 163.6

Table S3. Multiple linear regression analyses of variables impacting EQ-VAS and EQ-5D-3L index scores.

**Independent variables**		**EQ-VAS score (n = 111)**		**EQ-5D-3L index score (n = 126)**	
		**Beta coefficient (SE)**	***P* value**	**Beta coefficient (SE)**	***P* value**
**Age**		–0.055 (0.200)	0.790	0.001 (0.003)	0.955
**Gender**	**Female**	(reference)		(reference)	
	**Male**	–5.831 (4.916)	0.238	–0.027 (0.061)	0.658
**Atrial fibrillation**	**No**	(reference)		(reference)	
	**Yes**	–11.776 (4.850)	**0.017**	–0.013 (0.060)	0.833
**Reperfusion treatment**	**No**	(reference)		(reference)	
	**Yes**	9.571 (4.583)	**0.039**	0.137 (0.058)	**0.021**
**Baseline mRS ≤ 2**	**No**	(reference)		(reference)	
	**Yes**	24.733 (10.744)	**0.023**	0.239 (0.131)	0.071
**Baseline NIHSS**		–1.611 (0.496)	**0.002**	–0.026 (0.006)	**<0.001**

EQ-VAS – EuroQoL visual analog scale, EQ-5D-3L – EuroQoL Five Dimensions Three Levels, SE – standard error, mRS – modified Rankin Scale, NIHSS – National Institutes of Health Stroke Scale.
